# The Precipitin Reaction in the Urine in Pneumonia

**Published:** 1918-11

**Authors:** 


***The Precipitin Reaction in the Urine in Pneumonia.*** By WILLIAM J. QUIGLEY. *Journal of Infectious Diseases,* Sept., 1918.

Avery and Dochez have shown that a soluble substance is formed in pneumococcal infections in man, being demonstrable in the urine and blood by precipitation with specific serum.

The author has made a study of the sputum for the type of infection, and of the urine for precipitin reactions, in a hundred cases of lobar pneumonia due to the pneumococcus. Cultures from the sputum were made on blood-agar plates or intraperitoneal mouse inoculations made of sputum; the organisms thus obtained were tested for bile solubility and for agglutination by specific serum. There were 33 instances of infection with pneumococci of type I, 36 of type II, 13 of type III, and 18 of type IV. The incidence of type in pneumococcus infections may be quite constant through different seasons and localities.

It appears that a large percentage of fatal cases of pneumonia have a substance in the urine capable of giving a specific precipitin reaction with anti-pneumococcus serum; but that the presence of this substance in the urine is not of great unfavorable prognostic value, as the mortality, when it could not be demonstrated, was only slightly lower than when it was present. In every instance in which a precipitin reaction occurred, it was specific for the serum corresponding to the type of organism, as determined by agglutination of a pneumococcus isolated from the sputum. When this reaction is present in the urine, it is an accurate, and at present the most simple and rapid, means of type determination, and is, according to the author's results, present in 81 o/o of cases due to type I, II and III infections, although not always sufficiently early to warrant neglect of the sputum examination for the type of infection.

